# Antiproliferative Properties and G-Quadruplex-Binding of Symmetrical Naphtho[1,2-b:8,7-b’]dithiophene Derivatives

**DOI:** 10.3390/molecules26144309

**Published:** 2021-07-16

**Authors:** Antonino Lauria, Gabriele La Monica, Alessio Terenzi, Giuseppe Mannino, Riccardo Bonsignore, Alessia Bono, Anna Maria Almerico, Giampaolo Barone, Carla Gentile, Annamaria Martorana

**Affiliations:** 1 Dipartimento di Scienze e Tecnologie Biologiche Chimiche e Farmaceutiche “STEBICEF”, University of Palermo, Viale delle Scienze—Ed. 17, 90128 Palermo, Italy; antonino.lauria@unipa.it (A.L.); gabriele.lamonica01@unipa.it (G.L.M.); alessio.terenzi@unipa.it (A.T.); alessia.bono01@community.unipa.it (A.B.); annamaria.almerico@unipa.it (A.M.A.); giampaolo.barone@unipa.it (G.B.); carla.gentile@unipa.it (C.G.); 2 Plant Physiology Unit, Department of Life Sciences and Systems Biology, University of Turin, Via Quarello 15/A, 10135 Turin, Italy; giuseppe.mannino@unito.it; 3 Department of Chemistry, Technical University of Munich, Lichtenbergstr. 4, 85747 Garching, Germany; riccardo.bonsignore@tum.de

**Keywords:** planar heterocyclic scaffold, molecular docking, synthesis, G-Quadruplex, h-Telo, c-MYC, antiproliferative effect

## Abstract

*Background:* G-quadruplex (G4) forming sequences are recurrent in telomeres and promoter regions of several protooncogenes. In normal cells, the transient arrangements of DNA in G-tetrads may regulate replication, transcription, and translation processes. Tumors are characterized by uncontrolled cell growth and tissue invasiveness and some of them are possibly mediated by gene expression involving G-quadruplexes. The stabilization of G-quadruplex sequences with small molecules is considered a promising strategy in anticancer targeted therapy. *Methods*: Molecular virtual screening allowed us identifying novel symmetric bifunctionalized naphtho[1,2-b:8,7-b’]dithiophene ligands as interesting candidates targeting h-Telo and c-MYC G-quadruplexes. A set of unexplored naphtho-dithiophene derivatives has been synthesized and biologically tested through in vitro antiproliferative assays and spectroscopic experiments in solution. *Results*: The analysis of biological and spectroscopic data highlighted noteworthy cytotoxic effects on HeLa cancer cell line (GI_50_ in the low μM range), but weak interactions with G-quadruplex c-MYC promoter. *Conclusions*: The new series of naphtho[1,2-b:8,7-b’]dithiophene derivatives, bearing the pharmacophoric assumptions necessary to stabilize G-quadruplexes, have been designed and successfully synthesized. The interesting antiproliferative results supported by computer aided rational approaches suggest that these studies are a significant starting point for a lead optimization process and the isolation of a more efficacious set of G-quadruplexes stabilizers.

## 1. Introduction

In recent years it has emerged that the binding to G-quadruplex DNA structures by small molecules could be a promising targeted anticancer strategy [1,2,3,4,5,6]. A G-quadruplex (G4) is a non-canonical arrangement that both DNA and RNA nucleic acids can adopt [7]. Formed only in guanine-rich sequences, it consists of π-π mediated assembly of guanine tetrads stacked on top of each other and connected by looping bases. These structures are held by the interactions formed between guanine residues, playing the alternative role of acceptors and donors of Hoogsteen hydrogen-bonds and by the presence of monovalent cations, usually, K^+^ or Na^+^, which form electrostatic interactions with the central negative cavity lead by carbonyl oxygen atoms (guanine O6) [4].

G-quadruplex structures are recurrent in the human genome, especially in telomers, but also in several promoter regions of oncogenes like c-KIT [8], c-MYC [9], k-RAS [10], B-RAF [11], BCL-2 [12], RET [13], with some of them giving rise to multiple adjacent G4s, e.g., c-*KIT*. The formation of this non-canonical structure is implicated in numerous crucial phases of cellular regulation: inhibition of telomerase activity or gene’s transcription, alteration of recombination and splicing [1]. In this light, G-quadruplex stabilization can result in a blockage of telomere elongation, transcription, translation and accordingly down-regulation of the corresponding gene, with the possible result to prevent uncontrolled cell growth [3,14]. Consequently, G4s are considered interesting targets in anticancer therapy [1,2,3].

From a structural point of view, a molecule acting as a G4 stabilizer is characterized by a planar and often symmetric ring system, with a central aromatic scaffold able to stack on the ending tetrads of the G4. Furthermore, in a small molecule designed as a G4-binder candidate, the presence of side chains with positively charged or protonable nitrogen atoms can strengthen the interaction with the G4 via insertion within its grooves [2,4,15].

**Figure 1:**
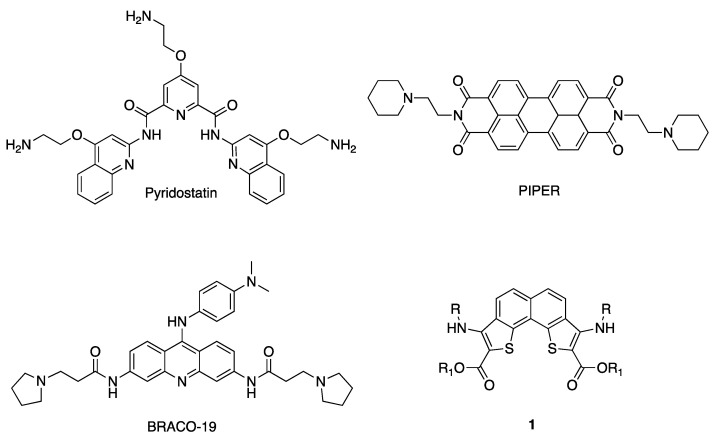
G-quadruplex stabilizers compounds and novel naphtho[1,2-b:8,7-b’]dithiophene ligands **1**.

A plethora of planar and symmetric compounds have been developed with this mechanism of action, including pyridostatin and analogues [16,17], perylene derivates (PIPER) [18], and acridine compounds (BRACO-19), (Figure 1) [19,20,21]. Some ligands have seen improved binding abilities when complexed with a transition metal as an alternative strategy [15,22,23,24].

Based on our continuous effort in the synthesis of bioactive planar heterocyclic ring systems, [25,26,27,28] and taking advantage of virtual screening results, we designed and synthesized novel symmetric bifunctionalized naphtho[1,2-b:8,7-b’]dithiophene (NDT) ligands **1** as potential G-quadruplex stabilizers (Figure 1). These compounds present ideal features for G4 binding as they show an extended aromatic planar area and protonable side chains. We investigated the in vitro antiproliferative activity of the synthesized molecules against HeLa cancer cell line and we tried to unravel the G4 binding capabilities of the naphtho[1,2-b:8,7-b’]dithiophene scaffold combining computational and experimental assays.

## 2. Results and Discussion

### 2.1. Computational Studies

With the aim to identify new G4 stabilizers, a large database of symmetrical and planar cores, symmetrically substituted, was built to conduct in silico structure-based studies.

The Virtual Screening Workflow implemented in Glide (Maestro suite) was applied to screen the in-house database against selected G-quadruplex sequences. The attention was focused on two of the most studied and characterized G4s, the c-MYC promoter and the h-Telo telomeric G4s [4], stored in Protein Data Bank (PDB, www.pdb.org, accessed on 1 June 2021).

In detail, the NMR-resolved structure of the c-MYC promoter G4 [5′-d(TGAGGGTGGGTAGGGTGGGTAA)-3′] complexed with the benzofuran derivative DC-34 (PDB id: 5w77) [20], and the h-Telo telomeric G4 (PDB id: 3CE5), a bimolecular human telomeric G-quadruplex sequence [5′-d(TAGGGTTAGGGT)-3′] co-resolved with BRACO-19 [21], were selected and appropriately prepared (see Section 3).

The protocol consisted of three steps (Glide High Through-put Virtual Screening (HTVS), Glide Standard Precision (SP) and Glide Extra Precision (XP) docking), able to skim rapidly the database and select the best ranked structures in each step (see Section 3).

**Figure 2:**
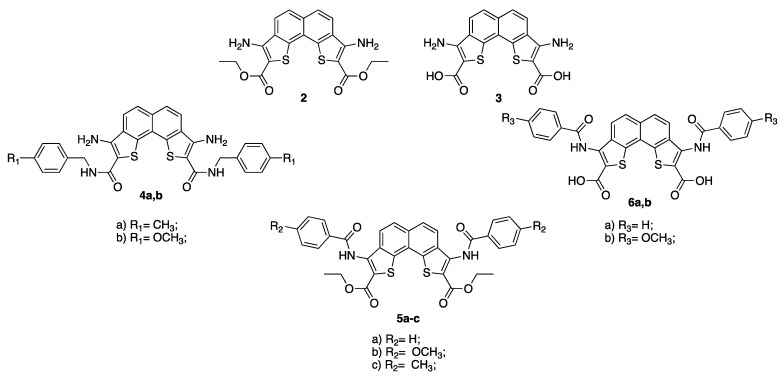
Structures of designed napthodithiophene compounds **2**–**6** as G4s stabilizers.

The analysis of the Glide XP docking data highlighted the naphtho[1,2-b:8,7-b’]dithiophene symmetric scaffold as interesting and unexplored heterocyclic ring system. This core resulted efficiently able to interact with the quartets of the selected G4 structures. All the best ranked NDT structures **2**–**6**, illustrated in Figure 2, confirmed the above mentioned pharmacophoric assumptions. The selected NDT molecules presented a central planar and aromatic core and side-moieties with exchangeable protons (amines and carboxylate). To reinforce the interactions with the G4 grooves, the NDT core was symmetrically derivatized with benzoyl portions (derivatives **4**–**6**).

Thus, to gain additional insight into the binding mode of the selected hits, these were submitted to further steps of structure-based studies (Induced Fit Docking, IFD), considering the flexibility of the selected G4s sequences.

#### Induced Fit Docking Analysis

The selected compounds were analyzed by means of Induced Fit Docking simulation within the chosen G4 structures. As reference compound, the symmetric perylene derivative PIPER (Figure 1), with proved c-MYC and h-Telo G4 stabilization capability [29,30,31], was docked applying the same virtual IFD protocol.

Table 1 shows the Induced Fit Docking results (Docking Scores, Prime Energy, and IFD Score) of the identified NDT molecules (Figure 2) and PIPER in complex with both c-MYC and h-Telo sequences.

Overall, all the selected NDT derivatives displayed IFD scores better than the well-known G4 stabilizer PIPER. This result could be due to the capability of the key central NDT core to stably stack over the guanine tetrads, forming strong π-π interactions with the nucleotide bases. Regarding the symmetrical functionalization of the NDT core with side moieties, both the free amines and the carboxamide groups efficiently form different H-bonds with the DNA bases, while the substituted benzoyl portions, oriented to the grooves, confirm their importance to further stabilize the ligand-nucleotide complex.

**Figure 3:**
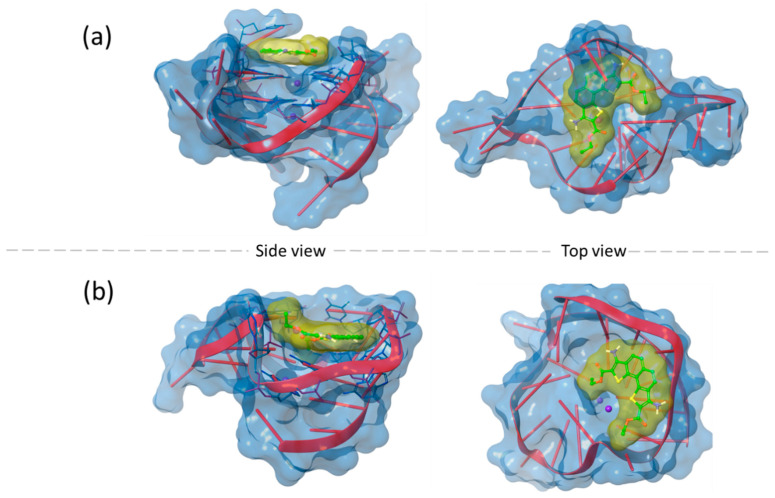
Molecular Docking Study of **2** in complex with h-Telo (PDB id: 3CE5) and c-MYC (PDB id: 5W77) G4 sequences; (**a**) side and top views obtained for the best pose of **2** in complex with the h-Telo G4 sequence, (**b**) side and top views obtained for the best pose of **2** in complex with the c-MYC G4 sequence.

In Figure 3, as an example, the best pose of the naphtho-dithiophene **2** in complex with both G4 sequences is considered. The side and the top views show a well-fitting of the NDT planar core, standing on top of the G4s, stacking over the guanines, and surrounding the central ion channel. These representations indicate that the NDT core, common to all the tested derivatives, is the key portion for the interaction with G4 structures.

**Figure 4:**
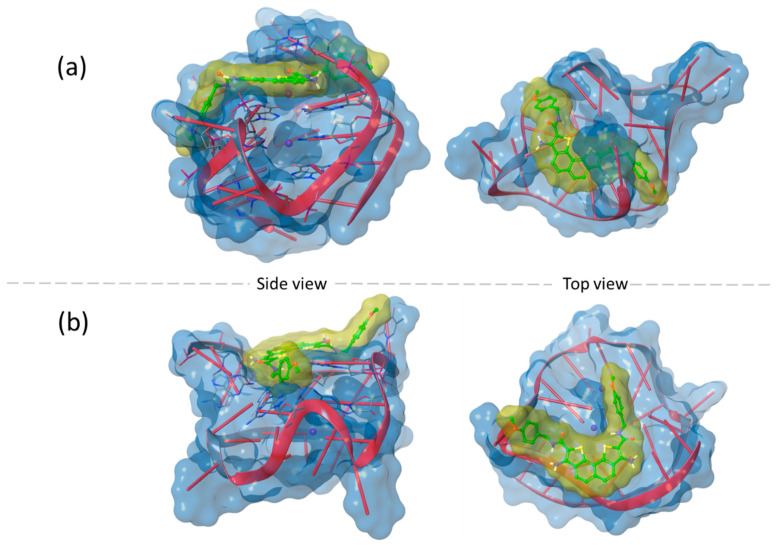
Molecular Docking Study of **4b** in complex with h-Telo (PDB id: 3CE5) and c-MYC (PDB id: 5W77) G4 sequences; (**a**) side and top views obtained for the best pose of **4b** in complex with the h-Telo G4 sequence; (**b**) shows the side and the top views obtained for the best pose of **4b** in complex with the c-MYC G4 sequence.

Interestingly, IFD scores suggest that the introduction of two side aromatic moieties in symmetric positions of the naphtho-dithiophene scaffold leads to reinforced groove binding capability. Among all, **4b**, whose best poses are reported in Figure 4, was the compound which gave the best G4 binding results, with IFD scores comparable or higher than the co-resolved ligands. Thus, based on the encouraging in-silico results, we decided to synthetize the selected NDT compounds.

### 2.2. Chemistry

**Scheme 1:**
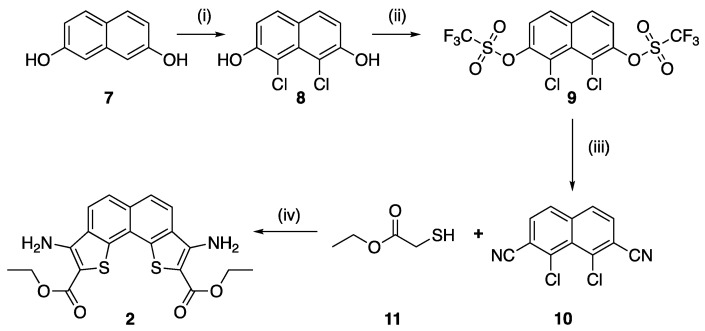
Synthesis of ethyl ester 3,8-diamino-naphtho[1,2-b:8,7-b’]dithiophene-2,9-carboxilate **2**. *Reagents and conditions*: (i) NCS, CH_3_CN, −15 °C, N_2_, (yield 86%); (ii) pyridine, Tf_2_O, 0 °C, (yield 75%); (iii) Zn(CN)_2_, Pd(PPh_3_)_4_, DMF, N_2_, (yield 69%); (iv) N(Et)_3_, DMSO, rt, (yield 73%).

Synthetic approaches for the preparation of the naphtho[1,2-b:8,7-b’]dithiophene ring system are rarely reported in the literature. As a cosequence, we planned a new synthetic strategy for the preparation of the naphtho-dithiophene series, as depicted in Scheme 1.

In detail, the 1,8-dichloro-2,7-naphtalenediol **8** was prepared in good yield (86%) from the reaction of 2,7-naphtalenediol **7** and N-chlorosuccinimide (NCS) under inert atmosphere and strict temperature control at −15 °C, to drive selectively the chlorination at C-1 and C-8 positions.

Compound **8** was then treated with trifluoromethanesulfonic anhydride to convert the 2,7-dihydroxy substituents into good leaving groups, (derivative **9**, yield 75%). The subsequent nucleophilic substitution with zinc cyanide in the presence of catalytic amount of tetrakis(triphenylphosphine), afforded 2,7-dicarbonitrilonaphthalene **10** (yield 69%). 

The presence of both CN and Cl substituents in vicinal positions to each other enhanced the aromatic nucleophilic substitution with ethyl thioglycolate **11**. Therefore, the in situ intramolecular cyclization and the consequent ring aromatization afforded the thiophene rings and the isolation of the title Naphtho-DiThiophene (NDT) ring system (compound **2**, yield 73%).

**Scheme 2:**
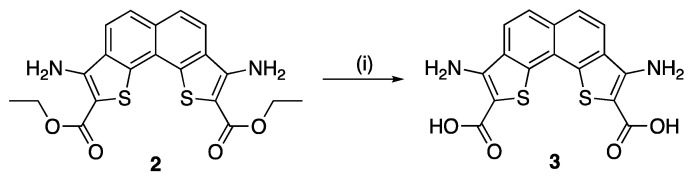
Synthesis of the Naphtho-DiThiophene **3**. *Reagents and conditions*: (i) 10% NaOH, EtOH, reflux, (yield 50%).

The ethyl-2,9-dicarboxilate functions of compounds **2** were hydrolyzed with sodium hydroxide to obtain the NDT analogue **3**, yield 50% (Scheme 2). These two naphtho-dithiophene derivatives rise from the virtual screening and could be used as lead compounds for the preparation of a new set of NDT derivatives.

**Scheme 3:**
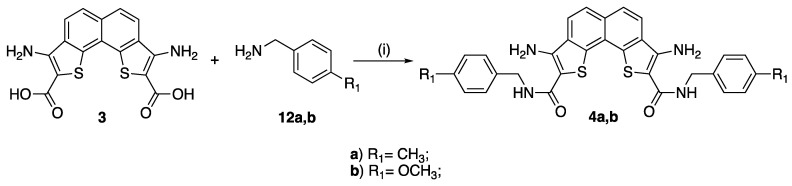
Synthesis of 3,8-diamino-N-benzyl-naphtho[1,2-b:8,7-b’]dithiophene-2,9-dicarboxamides (**4a**,**b**). *Reagents and conditions*: (i) HBTU, N(Et)_3_, AcOEt, (yields: **4a** = 43%; **4b** = 36%).

Symmetrical dicarboxylic compound **3** was subjected to reaction with appropriate benzylamines **12a**,**b**, in the presence of (2-(1H-benzotriazol-1-yl)-1,1,3,3-tetramethyluronium hexafluorophosphate (HBTU), (Scheme 3).

The amidation of the carboxylic groups afforded the isolation of naphtho-dithiophenes **4a**,**b**, owning the pivotal requirements for a G4 stabilizer: a symmetrical π-delocalized ring system, suitable for the π-π top-stacking interactions, and two side moieties with amines groups able to bind to the grooves of the G4. Furthermore, the steric bulk of the benzyl portions could selectively drive the compounds **4a**,**b** to stabilize G4s, preventing compound intercalation within the double-stranded DNA.

**Scheme 4:**
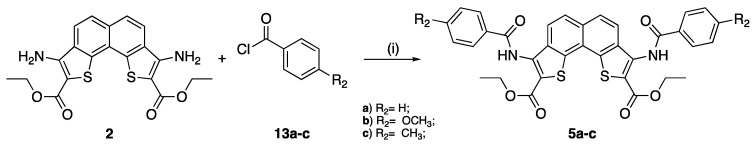
Synthesis ethyl 3,8-(benzoylamino)-naphtho[1,2-b:8,7-b’]dithiophene-2,9-dicarboxylates **5a**–**c**. *Reagents and conditions*: (i) pyridine, rt for 12h, (yields: **5a** = 24%; **5b** = 30%; **5c** = 23%).

Similar structural features were also introduced in derivatives **5a**–**c** by an alternative functionalization of the side groups, (Scheme 4). To obtain these compounds, the symmetrical amino derivative **2** was treated with substituted benzoyl chlorides **13a**–**c** with pyridine acting both as solvent and base promoting the nucleophilic substitution. Notwithstanding, any other reaction conditions led to a significant decrease in yields with a substantial recovery of starting material.

**Scheme 5:**
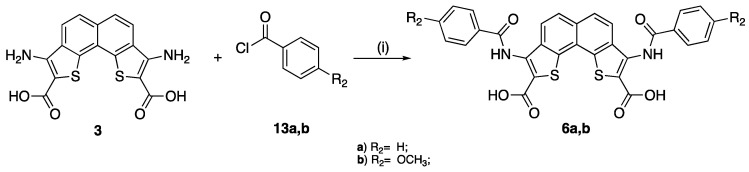
Synthesis 3,8-(benzoylamino)-naphtho[1,2-b:8,7-b’]dithiophene-2,9-dicarboxylic acid **6a**,**b**. *Reagents and conditions*: (i) pyridine, rt for 12h, (yields for both **6a**,**b** = 20%).

The same reaction conditions were adopted for the synthesis of dicarboxylic NDT compounds **6a**,**b**, starting from derivative **3** (Scheme 5).

From a chemical point of view, the symmetrical functionalization of the naphthodithiophene scaffold led to a general significant decrease in yields, probably due to the reduction of the reactivity of the central core after the reaction of the first functional group.

### 2.3. Biological Activity

#### In vitro Antiproliferative Activity

**Figure 5:**
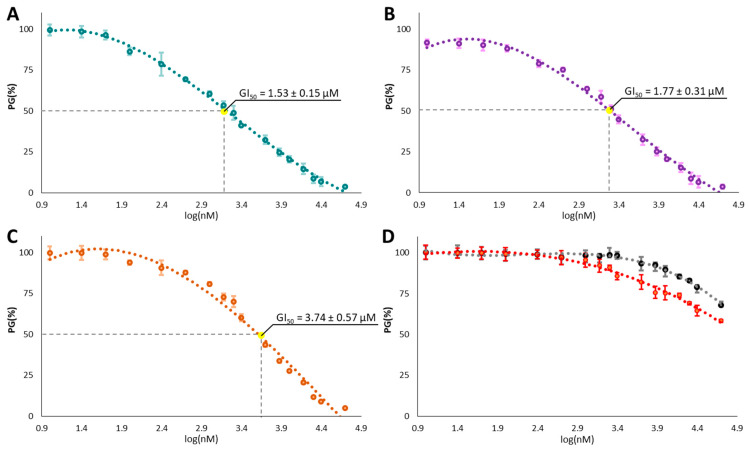
Antiproliferative activity exerted by the naphtho[1,2-b:8,7-b’]dithiophenes: (**A**) compound **2**; (**B**) derivative **4b**; (**C**) compound **5a**; (**D**) compound **3** (black line) and compound **4a** (red line) against HeLa cell line. In each panel, the antiproliferative activity is reported as GI_50_ value.

The antiproliferative activity of NDT derivatives was tested using the MTT assay on HeLa cell line. The tested compounds were incubated for 48 h using a concentration range between 0.01 and 50 μM. The NDT compounds **2**, **4b**, and **5a** revealed concentration-dependent antiproliferative activity (Figure 5) with GI_50_ values in the low micromolar range (Table 2).

Concerning the symmetrical functionalization of the NDT core with 3,8-diamine groups, the ethyl carboxylate ester derivative **2** showed a remarkable antiproliferative activity with a GI_50_ = 1.53 μM (Figure 5A), while the hydrolysis of the ester moieties to the corresponding carboxylic acid (NDT **3**) resulted in a detrimental effect with a complete loss of activity (Figure 5D, black line).

Symmetrical amidation of the carboxylic moieties produces different results based on the substituents on the benzyl ring. Indeed, while the 4-methoxy substituted 2,9-dicarboxamides derivatives **4b**, showed strong activity (Figure 5B), with GI_50_ comparable to the NDT compound **2**, the corresponding 4-methyl substituted derivative **4a**, had no inhibitory effects on tumor cell growth.

Finally, good activity was found for the 3,8-(benzoylamino) NDT compound, **5a** (Figure 5C) with GI_50_ of 3.74 μM.

### 2.4. Spectroscopic Studies in Solution

The NDT compound **2**, showing the best cytotoxic activity so far, was then tested for its G4 binding ability in solution using UV-Vis and circular dichroism (CD) titrations. Furthermore, a Förster Resonance Energy Transfer (FRET) melting assay was employed to verify its G4 stabilization properties. For these studies we have selected the G4 forming sequence of the c-MYC promoter, which is known to form a parallel G4 with the ending tetrad exposed for stacking interactions, as also showed in our docking calculations.

**Figure 6:**
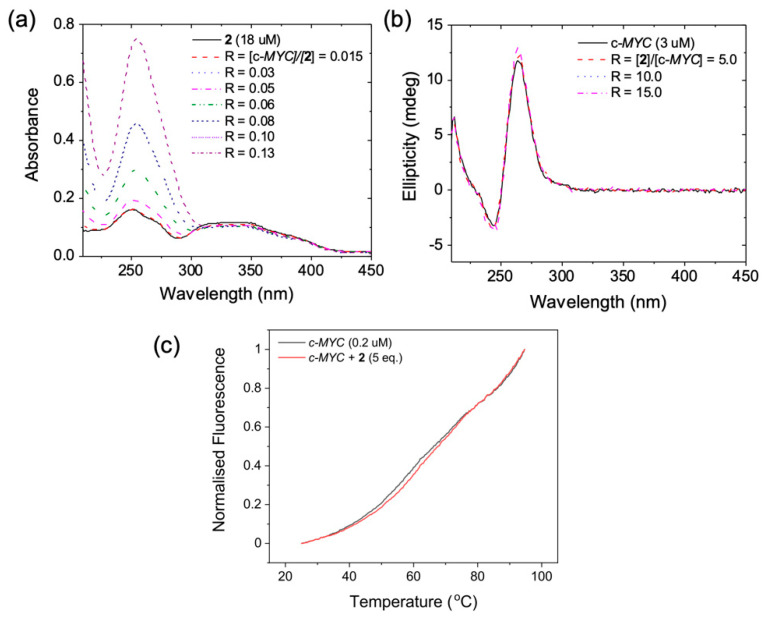
(**a**) UV-Vis spectra of **2** (black continuous line) in the presence of increasing amounts of c-MYC G4 collected in 100 mM KCl and 50 mM Tris-HCl buffer (pH = 7.4). (**b**) Circular dichroism spectra of (**a**) c-MYC G4 in presence of increasing amounts of **2** in 100 mM KCl and 50 mM Tris-HCl buffer (pH = 7.4). (**c**) Representative FRET DNA melting spectra of fluorolabelled c-MYC G4 alone (black curve) and in the presence of 5 equivalents of **2** (red trace) in 10mM potassium cacodylate buffer.

As observed in Figure 6, the characteristic band of compound **2** centered around 350 nm (black solid line), was only slightly affected (hypocromic effect) by the addition of increasing amounts of the c-MYC G4 oligonucleotide, indicating a minor binding in these experimental conditions. Circular dichroism measurements revealed that the typical character of the secondary c-MYC structure, characterized by a positive maximum at 264 nm and a negative minimum at 240 nm, is preserved upon interaction with our lead NDT compound **2**. Furthermore, it can be observed that the ellipticity at 264 nm slightly increases upon compound binding, accounting for an interaction between the two species in solution.

FRET melting experiments using the c-MYC G4 sequence properly labelled (see Section 3), further confirmed these spectroscopical results, pointing out a scarce stabilisation of the DNA secondary structure by **2**, with only ca. 1 °C increase of the melting temperature of the G4 in the presence of 5 Equation of the NDT derivative.

## 3. Materials and Methods

### 3.1. Computational Structure-Based Studies Experimental

#### 3.1.1. Ligand Preparation

The ligands and the G4 sequence–ligand complex used for the in silico studies were prepared as follows. The default setting of the LigPrep tool implemented in Schrödinger’s software (Version 2021-2, New York, NY, USA) was used to prepare the ligands for docking [32]. All possible tautomers and the combination of stereoisomers were generated at pH 7.0 ± 0.4 using the Epik ionization method. Energy minimization was subsequently performed using the integrated OPLS 2005 force field [33].

#### 3.1.2. Macromolecules Preparation

The crystal structure of a bimolecular parallel-stranded human telomeric G4 (PDB id 3CE5) [34] and the NMR resolved structure of the MYC G4 (PDB id: 5W77) [35] were downloaded from the Protein DataBank (PDB) [36,37]. The Protein Preparation Wizard of Schrödinger software was subsequently employed for further preparations of the G4 structures using the default settings [38,39]. Bond orders were assigned, and hydrogen atoms, as well as protonation of the heteroatom states were added using the Epik-tool (with the pH set at biologically relevant values, i.e., at 7.0 ± 0.4). The H-bond network was then optimized. The structures were subjected to a restrained energy minimization step (the RMSD of the atom displacement for terminating the minimization was 0.3 Å), using the Optimized Potentials for Liquid Simulations (OPLS) 2005 force field [33].

#### 3.1.3. Docking Validation

Molecular docking was performed by the Glide program [40,41,42]. The grid preparation was performed by assigning the original ligand as the centroid of the grid box. The generated 3D conformers were docked into the G4 sequence model using three different levels of precision sequentially (HTVS, High Throughput Virtual Screening; SP, Standard Precision; XP, Extra Precision) as the scoring functions. The proposed docking procedure was validated by the re-dock (XP mode) of the original co-resolved ligands within the binding nucleotides of 3CE5 and 5W77 by Glide XP docking. The results obtained were in good agreement with the experimental poses, showing an RMSD of 0.71 and 0.83, respectively.

#### 3.1.4. Induced Fit Docking

Induced fit docking simulation was performed using the IFD application as available [43,44] in the Schrödinger software suite (release 2021-2) [45], which is demonstrated to be an accurate and robust method to account for both ligand and G4 flexibility [46]. The IFD protocol was carried out as follows [47]: the ligands were docked into the rigid G4 models with scaled-down Van der Waals (VdW) radii. The Glide Extra Precision (XP) mode is used for the docking [40,41,42], and 20 ligand poses are retained for G4 structural refinements. The docking boxes were defined to include all the nucleotides within the dimensions of 25 Å × 25 Å × 25 Å from the center of the original ligands; the induced-fit G4–ligand complexes were generated using the Prime software [48,49]. The 20 structures from the previous step were submitted to the backbone refinements. All nucleotides with at least one atom located within 5.0 Å of each corresponding ligand pose were included in the refinement by Prime. All the poses generated were then hierarchically classified, refined, and further minimized into the active site grid before being finally scored using the proprietary GlideScore function, defined as follows in Equation (1):

(1) GScore=0.065 vdW +030 Coul + Lipo + Hbond + Metal + BuryP + RotB + Site

where: VdW is the van der Waals energy term, Coul is the Coulomb energy, Lipo is a Lipophilic contact term which rewards favorable hydrophobic interactions, Hbond is an H-bonding term, Metal is a metal-binding term (where applicable), BuryP is a penalty term applied to buried polar groups, RotB is a penalty for freezing rotatable bonds and Site is a term used to describe favorable polar interactions in the active site. 

Finally, the IFD score which accounts for both G4–ligand interaction energy and total energy of the system, was calculated (Equation (2)) and used to rank the IFD poses considering that the more negative is the IFDscore, the more favorable is the binding.

(2) IFD score = 1.0 Glide_Gscore + 0.05 Prime_Energy

### 3.2. Chemistry

#### 3.2.1. General Information

Unless otherwise indicated, all reagents and solvents were purchased from commercial sources and used without further purification. All melting points (°C) were determined on a Tottoli capillary apparatus (Büchi) and are uncorrected; IR spectra were determined in bromoform with a FT/IR 5300 spectrophotometer (Jasco). ^1^H-NMR and ^13^C-NMR spectra were respectively recorded, at 200 and 50.3 MHz in CDCl_3_ or DMSO-d_6_ solution, using an AC-E series 200 MHz spectrometer (Bruker). Chemical shifts values are given in ppm and referred as the internal standard to tetramethylsilane (TMS). The following abbreviations are used: br s = broad signal, s = singlet, d = doublet, t = triplet, q = quartet, m = multiplet, rt = room temperature. The purity of all compounds screened in biological assays was determined to be >95% by HPLC/MS analysis. Thin layer chromatography was performed on precoated (0.25 mm) silica gel GF254 plates, compounds were detected with 254 nm UV lamp. Column chromatography was performed with silica gel ASTM (230 and 400 mesh, Merck), or with a FLASH40i chromatography module (prepacked cartridge system, Biotage).

#### 3.2.2. Experimental Procedures and Product Characterization

##### Synthesis of 1,8-Dichloro-2,7-naphtalenediol (**8**)

A suspension of N-chlorosuccinimide (833.2 mg, 6.24 mmol) in dry acetonitrile was added to a solution of 2,7-naphtalenediol **7** (499.2 mg, 3.12 mmol) in dry acetonitrile (15 mL), at −15 °C and under inert atmosphere. The mixture was stirred, at rt for about 12 h, and then the solvent was evaporated under reduced pressure. The crude was purified by column chromatography on silica gel, using petroleum ether: ethyl acetate (*v*/*v*, 5:1) as eluent. Compound **8** was obtained as light-sensitive white needles (609.8 mg). Yield 86%. Mp 188–189 °C. IR ν_max_: 3450 cm^−1^. ^1^H-NMR (CDCl_3_) δ: 6.39 (br s, 2H, OH), 7.18 (d, *J* = 8.0 Hz, 2H, H-3, H-6) 7.65 (d, *J* = 8.0 Hz, 2H, H-4, H-5). ^13^C-NMR (DMSO-*d*_6_) δ: 110.4, 115.2, 124.8, 129.1, 153.7, 179.4. ESI-HRMS calcd for C_10_H_6_Cl_2_O_2_ [M + H]^+^: 227.9745, [M + H]^+^: 227.9750.

##### Synthesis of 1,1,1-Trifluoromethanesulfonic Acid-1,1′-(1,8-dochloro-2,7-naphatlendiyl) Ester (**9**)

To a mixture of **8** (458.1 mg, 2 mmol) and pyridine (0.94 mL, 11.6 mmol) in dry dichloromethane (15 mL), trifluoromethanesulfonic anhydride (0.74 mL, 4.5 mmol) was slowly added at 0 °C. After stirring for 4 h at rt, water (10 mL) and hydrochloric acid (1 M, 10 mL) were added. The resulting mixture was extracted with dichloromethane (30 mL × 3) and the combined organic layer was dried with Na_2_SO_4_ and concentrated in vacuo. The crude was purified by column chromatography in silica gel, using petroleum ether: ethyl acetate (*v*/*v*, 5:1) as eluent, to give **9** as white solid (739.7 mg). Yield 75%. Mp 120–121 °C. ^1^H-NMR (DMSO-*d*_6_) δ: 7.98 (d, *J* = 8.0 Hz, 2H, H-4, H-5), 8.40 (d, *J* = 8.0 Hz, 2H, H-3, H-6). ^13^C-NMR (DMSO-*d*_6_) δ: 121.2, 122.5, 123.3, 127.5, 131.6, 134.2, 146.3. ESI-HRMS calcd for C_12_H_4_Cl_2_F_6_O_2_S_2_ [M + H]^+^: 491.8730, [M + H]^+^: 491.8736.

##### Synthesis of 2,7-Dicarbonitrile-1,8-dichloronaphthalene (**10**)

To a degassed solution of **9** (493 mg, 1 mmol) in dry N,N-dimethylformamide (5 mL), under inert atmosphere, was added zinc cyanide (2.2 mmol) and tetrakis-(triphenylphosphine) palladium (155.4 mg, 0.1 mmol). The mixture was stirred for 12 h at 80 °C, then cooled to rt, diluted with ethyl acetate (50 mL) and poured into a saturated NaHCO_3_ solution. The white precipitated was filtered off, and the organic layer of filtrate was washed with water, extracted, dried with Na_2_SO_4_ (50 mL), and concentrated in vacuo. The obtained crude was purified by column chromatography in silica gel, using petroleum ether: ethyl acetate (*v*/*v*, 5:1) as eluent, to give **10** as white solid (170.5 mg). Yield 69%. Mp 253–254 °C. IR ν_max_: 2360 (CN) cm^−1^. ^1^H-NMR (DMSO-*d*_6_) δ: 8.16 (d, *J* = 8.0 Hz, 2H, H-4, H-5), 8.32 (d, *J* = 8.0 Hz, 2H, H-3, H-6).^13^C-NMR (DMSO-*d*_6_) δ: 110.3, 115.0, 124.3, 130.2, 131.0, 134.9, 153.8. ESI-HRMS calcd for C_12_H_4_Cl_2_N_2_ [M + H]^+^: 245.9751, [M + H]^+^: 245.9749.

##### Synthesis of Ethyl 3,8-Diaminonaphtho[1,2-b:8,7-b’]dithiophene-2,9-carboxylate (**2**)

To a stirred mixture of triethylamine (0.65 mL, 4.7 mmol) in dry DMSO (3 mL), ethyl thioglycolate **11** (0.3 mL, 2.67 mmol) was added. After stirring at rt for 20 min, a solution of **10** (220 mg, 0.89 mmol) in dry DMSO (3 mL) was added dropwise. The reaction was stirred at room temperature for further 3 h and then was poured onto water/ice. The precipitate was collected by filtration and dried to give orange-red solid of **2** (269.3 mg), crystallized from ethanol. Yield 73%. Mp 241–242 °C. IR ν_max_: 3495, 3367, 1659 cm^−1^. ^1^H-NMR (DMSO-*d*_6_) δ: 1.38 (t, *J* = 6.0 Hz, 6H, CH_3_ × 2), 4.37 (q, *J* = 6.0 Hz, 4H, CH_2_ × 2), 7.33 (br s, 4H, NH_2_ × 2), 8.08 (d, *J* = 8.7 Hz, 2H, H-5, H-6), 8.37 (d, *J* = 8.7 Hz, 2H, H-4, H-7). ^13^C-NMR (DMSO-*d*_6_) δ: 14.6, 60.0, 96.7, 99.5, 121.6, 125.3, 129.3, 133.6, 134.6, 150.2, 164.3. ESI-HRMS calcd for C_20_H_18_N_2_O_4_S_2_ [M + H]^+^: 414.0707, [M + H]^+^: 414.0714.

##### Synthesis of 3,8-Diaminonaphtho[1,2-b:8,7-b’]dithiophene-2,9-dicarboxylic Acid (**3**)

To a suspension of **2** (0.9 mmol) in ethanol (15 mL), a solution of NaOH 10% (5 mL) was added. The reaction mixture was heated to reflux for 3 h, and then, after cooling, HCl 6 N was added. The precipitate was collected by filtration, washed and dried to give **3** (161.3 mg). Yield 50%. Mp 268–269 °C. IR ν_max_: 3450, 3331, 1627 cm^−1^. ^1^H-NMR (DMSO-*d*_6_) δ: 6.66 (bs s, 4H, NH_2_ × 2), 7.92 (d, *J* = 8.0 Hz, 2H, H-5, H-6), 8.05 (d, *J* = 8.0 Hz, 2H, H-4, H-7). ^13^C-NMR (DMSO-*d*_6_) δ: 99.5, 113.8, 119.7, 123.8, 130.3, 131.1, 131.9, 142.9, 169.0. ESI-HRMS calcd for C_16_H_10_N_2_O_4_S_2_ [M + H]^+^: 358.0081, [M + H]^+^: 358.0075.

##### Synthesis of 3,8-Diamino-N-benzylnaphtho[1,2-b:8,7-b’]dithiophene-2,9-dicarboxamides (**4a**,**b**)

To a solution of acid **3** (1.0 mmol), in ethyl acetate (15 mL), HBTU (2.2 mmol) and triethylamine (3 mmol) were added. The mixture was stirred at rt for 30 min, and the appropriate benzylamine **12a**,**b** (2.0 mmol) was added. The reaction was allowed to proceed for further 12 h stirred at rt. The solvent was evaporated and the crude was purified by column chromatography in silica gel, using petroleum ether ethyl acetate as eluent. 

**4a**: Yield 43%. Mp 256–257 °C. ^1^H-NMR (DMSO-*d*_6_) δ: 2.28 (s, 6H, CH_3_), 4.43 (d, *J* = 6.0 Hz, 4H, CH_2_), 7.12-7.28 (m, 12H, H-2′, H-6′, H-3′, H-5′, NH_2_), 8.08 (d, *J* = 8.0 Hz, 2H, H-5, H-6), 8.28 (d, *J* = 8.7 Hz, 2H, H-4, H-7), 8.38 (t, *J* = 6.0 Hz, 2H, NH). ^13^C-NMR (DMSO-*d*_6_) δ: 20.6, 42.1, 100.8, 127.4, 128.7, 130.2, 132.3, 135.7, 137.0, 145.8, 147.1, 149.9, 151.0, 153.9, 164.9. ESI-HRMS calcd for C_32_H_28_N_4_O_2_S_2_ [M + H]^+^: 564.1653, [M + H]^+^: 564.1660.

**4b**: Yield 36%. Mp 259–260 °C. ^1^H-NMR (DMSO-*d*_6_) δ: 3.73 (s, 6H, OCH_3_), 4.40 (d, *J* = 2.0 Hz, 4H, CH_2_), 6.90 (d, *J* = 8.0 Hz, 4H, H-3′, H-5′), 7.18 (br s, 4H, NH_2_), 7.30 (d, *J* = 8.0 Hz, 4H, H-2′, H-6′), 8.07 (d, *J* = 8.7 Hz, 2H, H-5, H-6), 8.28 (d, *J* = 8.7 Hz, 2H, H-4, H-7), 8.36 (t, *J* = 2.0 Hz, 2H, NH). ^13^C-NMR (DMSO-*d*_6_) δ: 43.9, 55.4, 100.8, 127.4, 129.0, 129.9, 132.3, 135.9, 138.0, 145.9, 147.3, 150.0, 151.1, 154.0, 165.0. ESI-HRMS calcd for C_32_H_28_N_4_O_4_S_2_ [M + H]^+^: 596.1551, [M + H]^+^: 596.1559.

##### Synthesis of Ethyl 3,8-(Benzoylamino)-naphtho[1,2-b:8,7-b’]dithiophene-2,9-dicarboxylates (**5a**–**c**), 3,8-(Benzoylamino)-naphtho[1,2-b:8,7-b’]dithiophene-2,9-dicarboxylic Acids (**6a**,**b**)

To a suspension of **2** or **3** (0.37 mmol) and pyridine (0.56 mmol) was added the appropriate benzoyl chloride **13a**–**c** (0.56 mmol). The reaction mixture was stirred at room temperature over about 12 h, and then poured onto stirred water/ice. The precipitate was collected by filtration, dried overnight. The crude was purified by column chromatography in silica gel, using petroleum ether: ethyl acetate as eluent. 

**5a**: Yield 24%. Mp 269–270 °C. IR ν_max_: 3607, 1687, 1679 cm^−1^. ^1^H-NMR (DMSO-*d*_6_) δ: 1.36 (t, *J* = 6.0 Hz, 6H, CH_3_), 4.38 (d, *J* = 6.0 Hz, 4H, CH_2_), 7.37–7.68 (m, 6H, C_6_H_5_), 7.82 (d, *J* = 8.1 Hz, 2H, H-5, H-6), 8.13 (d, *J* = 8.1 Hz, 2H, H-4, H-7), 8.19–8.36 (m, 4H, C_6_H_5_), 10.71 (s, 2H, NH). ^13^C-NMR (DMSO-*d*_6_) δ:14.3, 62.3, 118.0, 119.9, 123.3, 124.3, 124.9, 127.9, 129.0, 132.8, 134.3, 134.8, 136,6, 141.0, 164.1, 165.3. ESI-HRMS calcd for C_34_H_26_N_2_O_6_S_2_ [M + H]^+^: 622.1232, [M + H]^+^: 622.1227.

**5b**: Yield 30%. Mp 276–277 °C. IR ν_max_: 3601, 1696, 1672 cm^−1^. ^1^H-NMR (DMSO-*d*_6_) δ: 1.38 (t, *J* = 6.0 Hz, 6H, CH_3_), 3.82 (s, 6H, OCH_3_), 4.37 (d, *J* = 6.0 Hz, 4H, CH_2_), 7.02 (d, *J* = 8.0 Hz, 4H, H-3′, H-5′), 7.90 (d, *J* = 8.0 Hz, 4H, H-2′, H-6′), 8.09 (d, *J* = 8.1 Hz, 2H, H-5, H-6), 8.39 (d, *J* = 8.1 Hz, 2H, H-4, H-7), 12.64 (s, 2H, NH). ^13^C-NMR (DMSO-*d*_6_) δ: 14.3, 55.3, 61.3, 113.5, 117.9, 119.8, 122.7, 124.7, 124.8, 127.1, 129.6, 134.8, 136.7, 141.1, 162.3, 164.5, 165.6. ESI-HRMS calcd for C_36_H_30_N_2_O_8_S_2_ [M + H]^+^: 682.1443, [M + H]^+^: 682.1437.

**5c**: Yield 23%. Mp 273–274 °C. IR ν_max_: 3606, 1700, 1677 cm^−1^. ^1^H-NMR (DMSO-*d*_6_) δ: 1.37 (t, *J* = 6.0 Hz, 6H, CH_3_), 2.40 (s, 6H, CH_3_), 4.28 (d, *J* = 6.0 Hz, 4H, CH_2_), 7.28 (d, *J* = 8.0 Hz, 4H, H-3′, H-5′), 7.41 (d, *J* = 8.0 Hz, 4H, H-2′, H-6′), 8.07 (d, *J* = 8.1 Hz, 2H, H-5, H-6), 8.41 (d, *J* = 8.1 Hz, 2H, H-4, H-7), 12.64 (s, 2H, NH). ^13^C-NMR (DMSO-*d*_6_) δ: 14.3, 21.4, 61.3, 117.8, 119.8, 122.6, 124.7, 124.8, 128.1, 129.6, 131.2, 134.9, 136.7, 141.1, 142.3, 164.5, 165.6. ESI-HRMS calcd for C_36_H_30_N_2_O_6_S_2_ [M + H]^+^: 650.1545, [M + H]^+^: 650.1539.

**6a**: Yield 20%. Mp 279–280 °C. 3603, 3130, 1695, 1636 cm^−1^ IR ν_max_: 3603, 3129, 1672, 1629 cm^−1^. ^1^H-NMR (DMSO-*d*_6_) δ: 7.59–7.77 (m, 6H, C_6_H_5_), 7.81 (d, *J* = 8.1 Hz, 2H, H-5, H-6), 8.11–8.25 (m, 6H, C_6_H_5_, H-4, H-7), 10.71 (s, 2H, NH). ^13^C-NMR (DMSO-*d*_6_) δ: 117.5, 119.3, 122.6, 124.6, 124.6, 127.8, 129.0, 132.2, 134.1, 135.0, 137.4, 141.5, 166.2, 169.2. ESI-HRMS calcd for C_30_H_18_N_2_O_6_S_2_ [M + H]^+^: 566.0606, [M + H]^+^: 566.0613.

**6b**: Yield 20%. Mp 282–283 °C. IR ν_max_: 3601, 3125, 1696, 1630 cm^−1^. ^1^H-NMR (DMSO-*d*_6_) δ: 3.89 (s, 6H, OCH_3_), 7.03 (d, *J* = 8.1 Hz, 2H, H-5, H-6), 7.15 (d, *J* = 8.0 Hz, 4H, H-3′, H-5′), 7.90 (d, *J* = 8.1 Hz, 2H, H-4, H-7), 8.08 (d, *J* = 8.0 Hz, 4H, H-2′, H-6′), 12.64 (s, 2H, NH). ^13^C-NMR (DMSO-*d*_6_) δ: 55.2, 113.6, 117.6, 119.3, 122.6, 124.6, 124.8, 127.1, 129.6, 135.0, 137.4, 141.6, 162.3, 165.9, 169.2. ESI-HRMS calcd for C_32_H_22_N_2_O_8_S_2_ [M + H]^+^: 626.0817, [M + H]^+^: 626.0823.

### 3.3. Biology

#### 3.3.1. Cell Culture

Human cervical cancer (HeLa), purchased from American Type Culture Collection (Rockville, MD, USA), were cultured in Roswell Park Memorial Institute (RPMI) medium supplemented with 5% fetal bovine serum (FBS), 2 mM L-glutamine, 50 IU/mL penicillin, and 50 μg/mL streptomycin. Before antiproliferative experiments, HeLa cells were trypsinized when their confluences reached 75–85%.

#### 3.3.2. Antiproliferative Activity

The antiproliferative activity of the selected compounds was performed in in vitro using 3-[4,5-dimethylthiazole-2-yl]-2,5-diphenyltetrazolium bromide (MTT) assay, as previously described **[50]**. Briefly, the cells were seeded into 96-well plates and incubated for 24 h at 5% CO_2_ and 37 °C. After this incubation time, the cell medium was discarded and replaced with an equal volume containing fresh medium supplemented by 5% (*v*/*v*) FBS and an opportune amount of the synthetized compounds. In particular, the synthetized molecules were previously solubilized in dimethyl sulfoxide (DMSO) in order to obtain a concentration equal to 20 mM, and then different dilutions (0.01–50 μM) were prepared in fresh medium and added in each well for 48 h. In each experiment, the concentration of DMSO never exceeded 0.25% (*v*/*v*) and cell treated simply with culture medium supplemented with 0.25% (*v*/*v*) DMSO were used as control. After the treatment time, fresh medium containing 0.5 mg/mL MTT reagent was added in each well, and then the plates were again incubated for 3 h at 5% CO_2_ and 37 °C. The alive cells in the wells metabolize MTT forming the formazan salt that is consequently dissolved in DMSO and spectrophotometrically monitored at 570 nm using a microplate reader (GloMax^®^ Multidetection Plate Reader, Promega^®^). Since the absorbance read at 570 nm is directly proportional to the number of living and metabolically active cells after the different treatments, the percentage of growth (PG%) with respect to untreated cell control for each synthetized molecule was calculated according to Equation (3) or Equation (4):

*If* (OD_test_ − OD_tzero_) ≥ 0, then (3)

*If* (OD_test_ − OD_tzero_) < 0, then (4)

where: OD_test_ is the average value related to the optical density measurements before exposure of cells to the test extract; OD_tzero_ is the average values related to the optical density measurements after the desired period of time; OD_ctr_ is the average values related to the optical density measurements after the desired period of time and with no exposure of cells to treatment. The concentration necessary to inhibit the 50% cell growth (GI_50_) for each synthetized compound was calculated using concentration—response curves and linear regression analysis by fitting the test concentrations that give PG values above and below the reference value. Each result is a mean value of five separate experiments.

### 3.4. Spectroscopic Studies

G4 forming 20-mer sequence from c-MYC promoter (5′-GGGAGGGTGGGGAGGGTGGG-3′) was purchased from IDT (Integrated DNA Technologies, Belgium) in HPLC purity grade. The oligonucleotide was suspended in MilliQ water to yield a 100 μM stock solution. Dilution to the desired concentration was performed using 50 mM Tris-HCl/100 mM KCl buffer (pH 7.4). The G4 folding was obtained by heating the solutions up to 90 °C for 5 min and then by slowly cooling down to room temperature overnight. Concentration of the DNA sequence solutions was checked measuring their absorbance and using the extinction coefficient provided by the manufacturer.

UV-vis spectra were collected on a Cary 100 double beam spectrophotometer, using 1 cm path-length quartz cuvettes. Titrations were carried out by adding increasing amounts of c-MYC stock solution to a solution of compound **2** with constant concentration.

Circular dichroism spectra were recorded on a J-715 spectropolarimeter (Jasco), using 1 cm pathlength quartz cuvettes, at 25 °C with the following parameters: step resolution: 0.2 nm, speed: 200 nm min^−1^, accumulations: 4, response: 0.5 s, bandwidth: 1 nm. Titrations were carried out by adding increasing amounts of **2** stock solution to a *MYC* solution with constant concentration. 

Stock solution of **2** was prepared in acetonitrile for both UV-Vis and CD experiments. The final percentage of acetonitrile never exceeded 5% in the final solutions. Time interval before each consecutive addition was of 5 min in both experiments.

### 3.5. FRET DNA Melting Assay

Föster resonance energy transfer (FRET) experiments were run on an Applied Biosystems^®^ QuantumStudio 5 Real-Time PCR thermocycler (Thermo Fisher Scientific, Waltham, MA, USA) equipped with a FAM filter (λ_ex_ = 492 nm; λ_em_ = 516 nm). The thermocycler was set to perform a stepwise increase of 0.3 °C every 30 s, from 25 °C to 95 °C, and measurements were acquired after each step. 

The oligonucleotide was purchased from Eurogentec (Belgium) in HPLC purity grade. The FRET probes used were 6-carboxyfluorescein (FAM) and 6-carboxytetramethylrhodamine (TAMRA). The lyophilized fluorolabelled pu27-mer c-MYC, d[TGGGGAGGGTGGGGAGGGTGGGGAAGG]_,_ (T_m_ = 66.20 °C), was firstly diluted in deionized water to obtain 100 μM stock solutions. Stock solutions were diluted to a concentration of 400 nM in potassium cacodylate buffer (10 mM, pH = 7.4), and then annealed to form G4 structures by heating to 95 °C for 5 min, followed by cooling to room temperature overnight. 

Experiments were carried out in triplicates in a 96-well plate with a total volume of 30 μL. The final concentration of the G4-oligonucleotide was set to 200 nM in potassium cacodylate buffer (10 mM, pH = 7.4). Stock solutions of NDT **2** in acetonitrile (1 mM) were freshly prepared prior to the experiments. The stock solutions were further diluted to a final concentration of 1 μM (with a total percentage of acetonitirle of approx. 0.1%) in potassium cacodylate buffer (10 mM, pH = 7.4) to achieve G4: **2** stoichiometry of 1: 5. 

To compare different sets of data, FAM emission was normalized (0 to 1). T_m_ is defined as the temperature at which the normalized emission is 0.5 and ΔT_m_ is defined as the difference of T_m_ between treated samples and untreated controls. Independent experiments were run in triplicate.

## 4. Conclusions

Computer-aided rational approaches allowed the identification of a new series of symmetrical planar heterocyclic compounds. The bifunctionalized naphtho[1,2-b:8,7-b’]dithiophene derivatives own several pharmacophoric features recurrent in small molecules with a proved stabilization effect on sequences able to fold in G-quadruplex arrangements. The computational analysis of the interaction between the designed ligands and two G4 forming sequences (h-Telo and c-MYC) highlighted an optimal stacking of the NDT scaffold on top of the guanine tetrads with the formation of π-π interactions between the DNA bases and the aromatic symmetrical core. The presence of two symmetrical side aromatic moieties fostered the binding of the complex with the G4 oligonucleotides. Both the amine groups and the carboxamide functions establish H-bonds with the nitrogen atoms of the DNA bases. Furthermore, bulky benzyl moieties reinforce the interactions with G4 grooves, suggesting a selective activity towards G4 DNA, in spite of the intercalation within the double-stranded DNA. The unexplored naphtho[1,2-b:8,7-b’]dithiophene derivatives, arising from the virtual screening, were successfully synthesized. In particular, the construction of the NDT scaffold was carried out thought the applications of new selective synthetic strategies.

To evaluate the antiproliferative effect of the synthesized compounds, in vitro cytotoxic assays were performed on HeLa cancer cell line. After treatment with NDT derivatives, interesting values of GI_50_ in the low μM range (GI_50_ = 1.53 μM and GI_50_ = 1.77 μM respectively for **2** and **4b**) were measured.

Spectroscopic studies in solution (UV-Vis, CD, and FRET melting assays) using compound **2** and the c-MYC promoter sequence as models of NDT and G4, respectively, were performed to have a first glimpse of what kind of interaction between the synthesized derivatives and G4 structures can subsists. Contrary to expectation, minor binding and a scarce stabilization of the selected DNA structure was observed, at least in our experimental settings. The encouraging antiproliferative effects and the favorable features as G4 binders of these compounds so far demand for further investigations and interaction studies using different G4s as well as different compounds at increasing concentrations.

In an attempt to improve the G4-biding properties of the naphtho[1,2-b:8,7-b’]dithiophene compounds, coordination to metals like Ni(II) and Pt(II) are currently considered and will be the subjects of new studies. This modification should result in an increased π-π interactions with the G-quartets as those observed by using porphyrin scaffolds [51,52,53,54]. This enhanced affinity towards the tetrads might very likely discourage any flanking bases binding, accounting instead for an increased G4-stabilisation.

## Figures and Tables

**Table 1:** Prime energy, Docking and Induced Fit Docking (IFD) scores of the studied compounds in complex with h-Telo and c-MYC G4 oligonucleotides.

	h-Telo G4			c-MYC G4		
Title	Docking Score	Prime Energy	IFD Score	Docking Score	Prime Energy	IFD Score
**2**	−7.124	−3437.0	−178.97	−4.855	−3254.1	−167.56
**3**	−5.162	−3460.1	−178.19	−4.683	−3239.2	−166.67
**4b**	−9.782	−3490.7	−184.32	−6.755	−3261.2	−169.82
**4a**	−8.480	−3450.3	−180.99	−6.365	−3266.3	−169.68
**5a**	−7.934	−3439.7	−179.92	−5.598	−3245.1	−167.86
**5c**	−8.695	−3426.5	−180.02	−6.711	−3231.0	−168.26
**5b**	−9.793	−3466.8	−183.13	−7.479	−3241.6	−169.56
**6a**	−7.441	−3470.5	−180.97	−4.093	−3260.4	−167.11
**6b**	−4.875	−3518.7	−180.81	−3.752	−3265.9	−167.05
Correlate ligand ^a,b^	−9.645	−3497.8	−184.74 ^a^	−5.399	−3267.9	−168.98 ^b^
PIPER	−8.272	−3393.2	−177.93	−7.433	−3175.8	−166.23

The correlate ligand: ^a^ BRACO-19; ^b^ DC-34.

**Table 2:** The antiproliferative activity of the tested NDT compounds (incubation time: 48 h) against HeLa cell lines expressed as GI_50_ values (GI_50_ ± SE (μM)).

Compound	GI_50_ (μM)
**2**	1.53 ± 0.15
**3**	>50
**4a**	>50
**4b**	1.77 ± 0.31
**5a**	3.74 ± 0.57

## Data Availability

The data used to the findings of this study are included within the article and the Supporting Information file.

## Supplementary Materials

The following are available online, S1: ^1^H NMR spectrum of ethyl ester 3,8-diamino-naphtho[1,2-b:8,7-b’]dithiophene-2,9-carboxilate **2**, S2: ^13^C NMR spectrum of ethyl ester 3,8-diamino-naphtho[1,2-b:8,7-b’]dithiophene-2,9-carboxilate **2**, S3: ^13^C DEPT spectrum of ethyl ester 3,8-diamino-naphtho[1,2-b:8,7-b’]dithiophene-2,9-carboxilate **2**, S4: ^1^H NMR spectrum of 3,8-diamino-naphtho[1,2-b:8,7-b’]dithiophene-2,9-dicarboxylic acid **3**, S5: ^13^C NMR spectrum of 3,8-diamino-naphtho[1,2-b:8,7-b’]dithiophene-2,9-dicarboxylic acid **3**, S6: ^1^H NMR spectrum of 3,8-diamino-N2,N9-bis(4-methylbenzyl)naphtho[1,2-b:8,7-b’]dithiophene-2,9-dicarboxamide **4a**, S7: 1H NMR spectrum of 3,8-diamino-N2,N9-bis(4-methoxybenzyl)naphtho[1,2-b:8,7-b’]dithiophene-2,9-dicarboxamide **4b**, S8: ^1^H NMR spectrum of diethyl 3,8-bis(4-methoxybenzamido)naphtho[1,2-b:8,7-b’]dithiophene-2,9-dicarboxylate **5b**, S9: ^1^H NMR spectrum of 3,8-bis(4-methoxybenzamido)naphtho[1,2-b:8,7-b’]dithiophene-2,9-dicarboxylic acid **6b**.

## Abbreviations

G-quadruplex (G4); Naphtho[1,2-b:8,7-b’]dithiophene (NDT); Protein Data Bank (PDB); Induced Fit Docking (IFD); Glide High Through-put Virtual Screening (HTVS); Glide Standard Precision (SP); Glide Extra Precision (XP); N-chlorosuccinimide (NCS); (2-(1H-benzotriazol-1-yl)-1,1,3,3-tetramethyluronium hexafluorophosphate (HBTU); Circular Dichroism (CD); Förster Resonance Energy Transfer (FRET).
