# Supplementary material for: Antiproliferative Properties and G-Quadruplex-Binding of Symmetrical Naphtho[1,2-b:8,7-b’]dithiophene Derivatives
Source: Molecules. 2021 Jul 16;26(14):4309. doi: 10.3390/molecules26144309 (PMC8303715; doi:10.3390/molecules26144309)
Supplement: Supplementary file 1 [file molecules-26-04309-s001.zip › molecules-1272578-supplementary.pdf]

Supporting Information

Antiproliferative properties and G-quadruplex-binding of symmetrical Naphtho[1,2-b:8,7-b']dithiophene derivatives

Antonino Lauria [1], Gabriele La Monica [1], Alessio Terenzi [1], Giuseppe Mannino [2], Riccardo Bonsignore [3], Alessia Bono [1], Anna Maria Almerico [1], Giampaolo Barone [1], Carla Gentile [1], Annamaria Martorana [1,*]

[1] Dipartimento di Scienze e Tecnologie Biologiche Chimiche e Farmaceutiche "STEBICEF" – University of Palermo, Viale delle Scienze – Ed. 17 -90128 Palermo, Italy; antonino.lauria@unipa.it (A.L.); gabriele.lamonica01@unipa.it (G.L.M.); alessio.terenzi@unipa.it (A.T.); alessia.bono01@community.unipa.it (A.B.); annamaria.almerico@unipa.it (A.M.A.); giampaolo.barone@unipa.it (G.B.); carla.gentile@unipa.it (C.G.); annamaria.martorana@unipa.it (A.M.)

[2] Department of Life Sciences and Systems Biology, Plant Physiology Unit, University of Turin, Via Quarello 15/A, 10135 Turin, Italy; giuseppe.mannino@unito.it (G.M.)

[3] Department of Chemistry, Technical University of Munich, Lichtenbergstr. 4, 85747 Garching Germany; riccardo.bonsignore@tum.de (R.B.)

* Correspondence: annamaria.martorana@unipa.it (A.M.); Tel.: +39 – 091 2389 6821

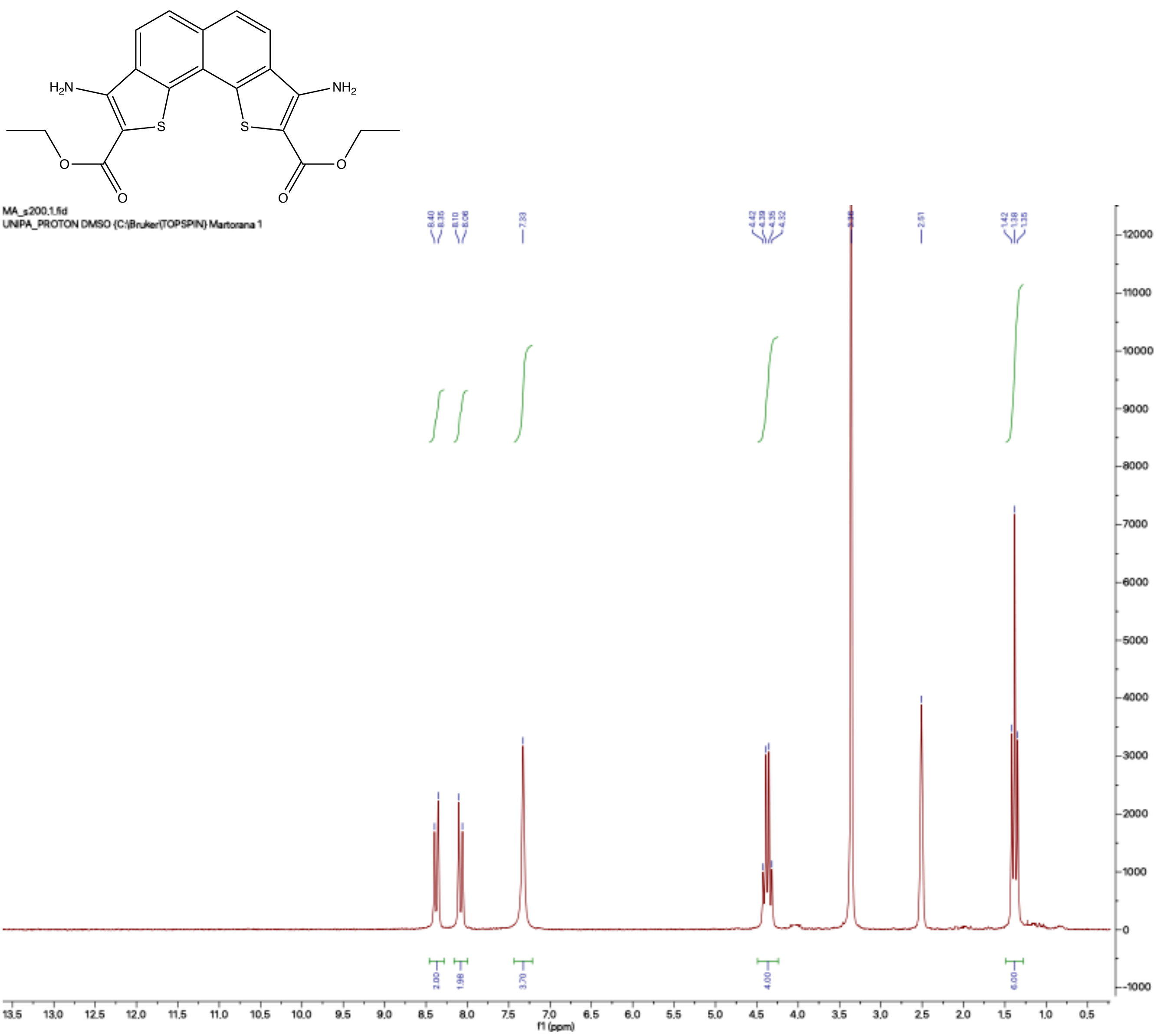

S1: ^{1}H NMR spectrum of ethyl ester 3,8-diamino-naphtho[1,2-b:8,7-b′]dithiophene-2,9-carboxilate **2.**

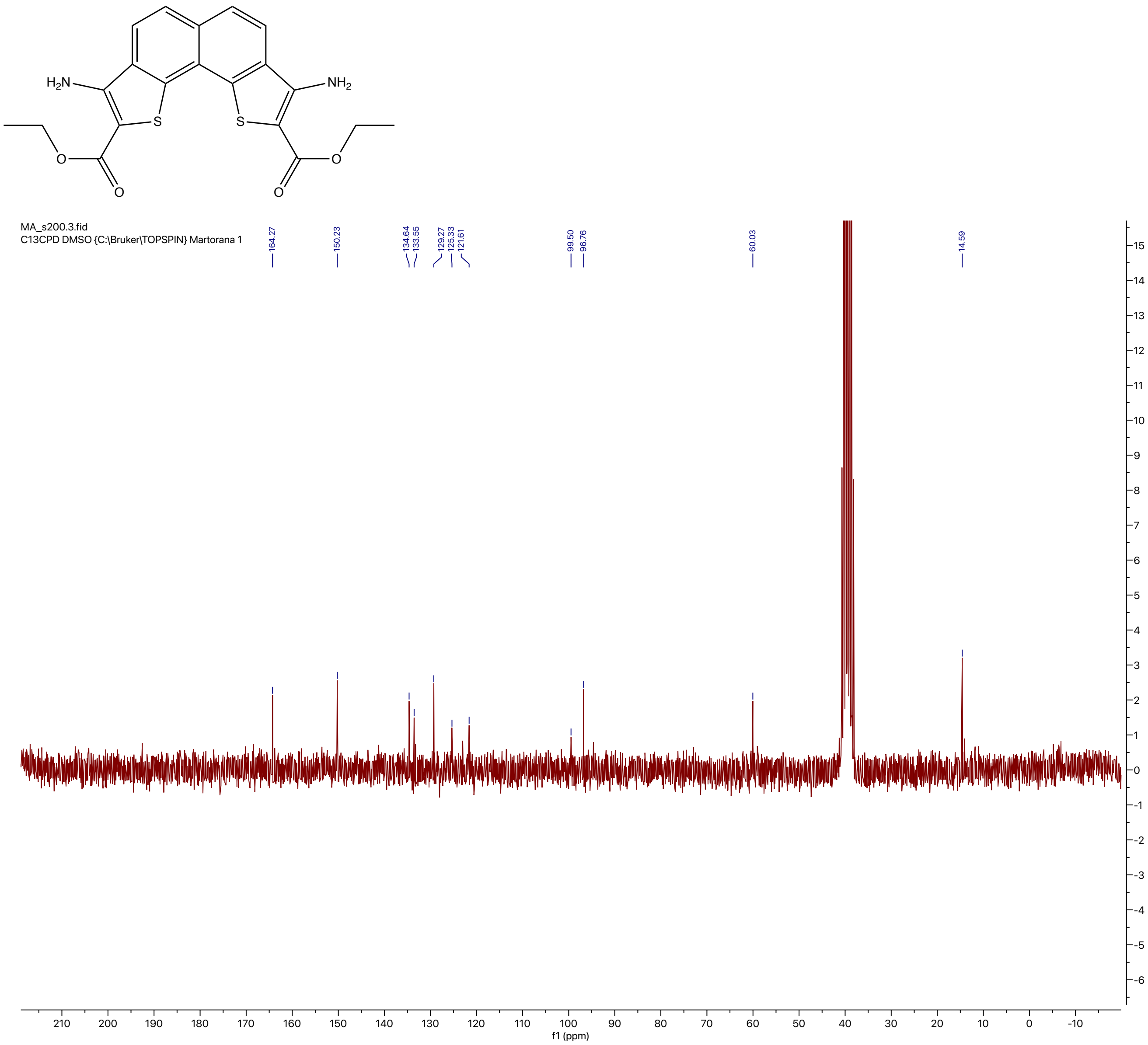

S2: ^{13}C NMR spectrum of ethyl ester 3,8-diamino-naphtho[1,2-b:8,7-b′]dithiophene-2,9-carboxilate **2.**

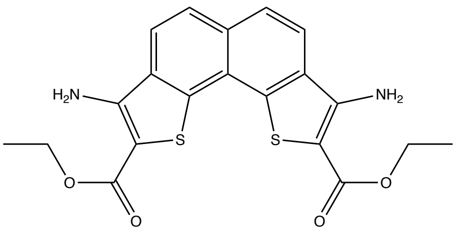

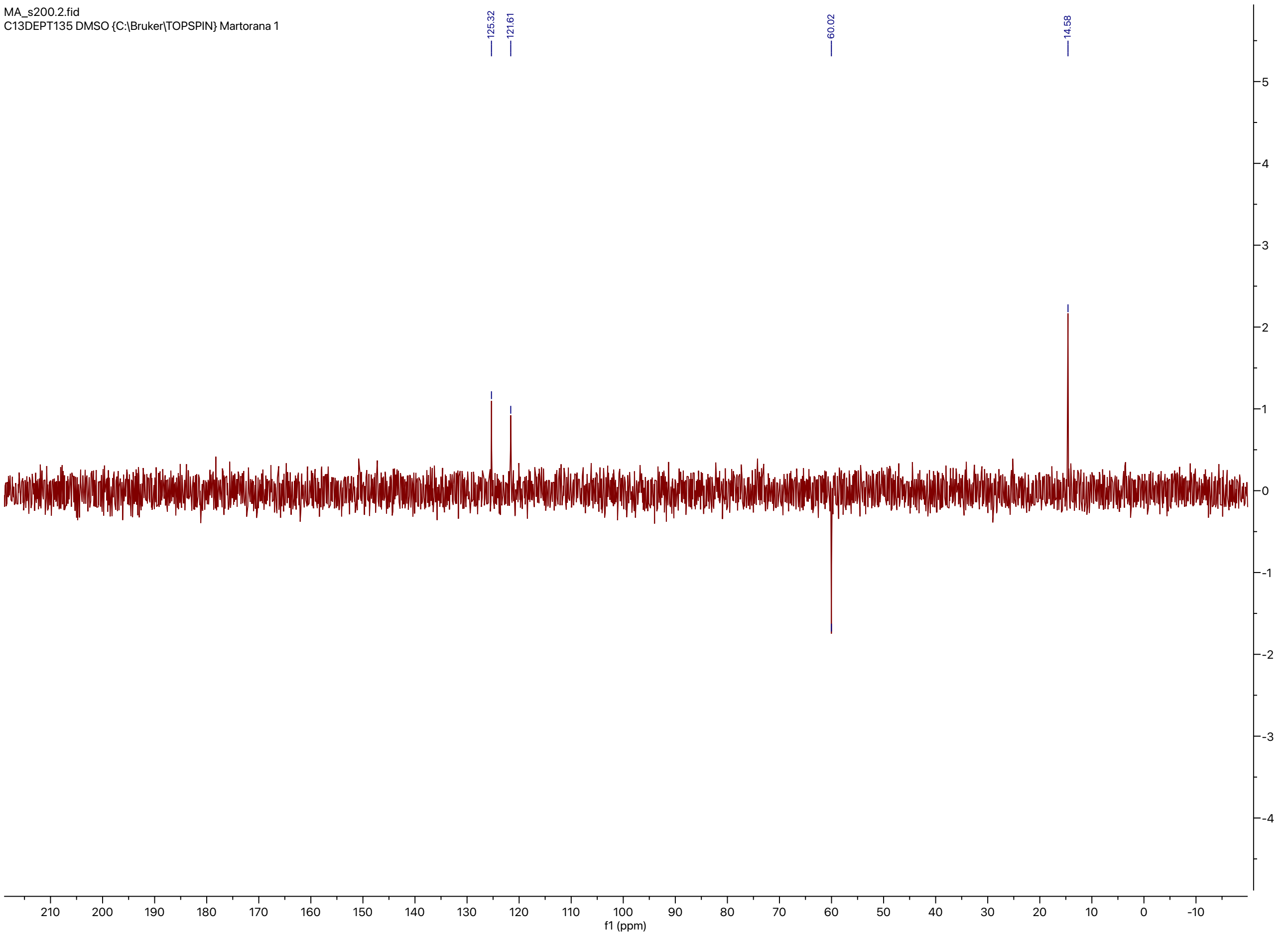

S3: ^{13}C DEPT spectrum of ethyl ester 3,8-diamino-naphtho[1,2-b:8,7-b′]dithiophene-2,9-carboxilate **2.**

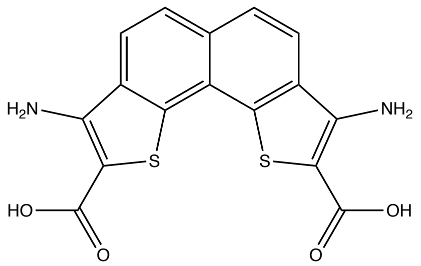

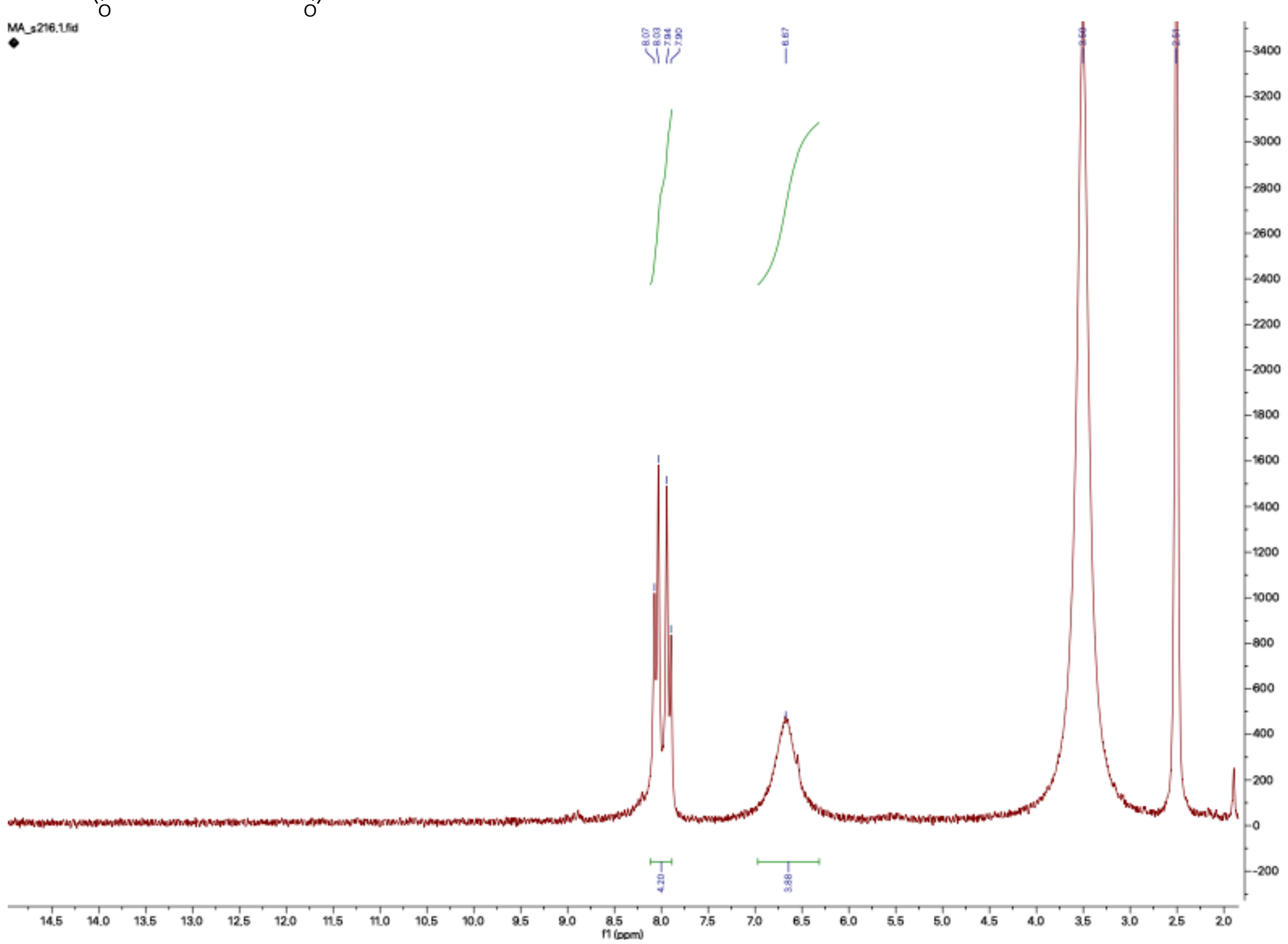

S4: ^{1}H NMR spectrum of 3,8-diamino-naphtho[1,2-b:8,7-b′]dithiophene-2,9-dicarboxylic acid **3**.

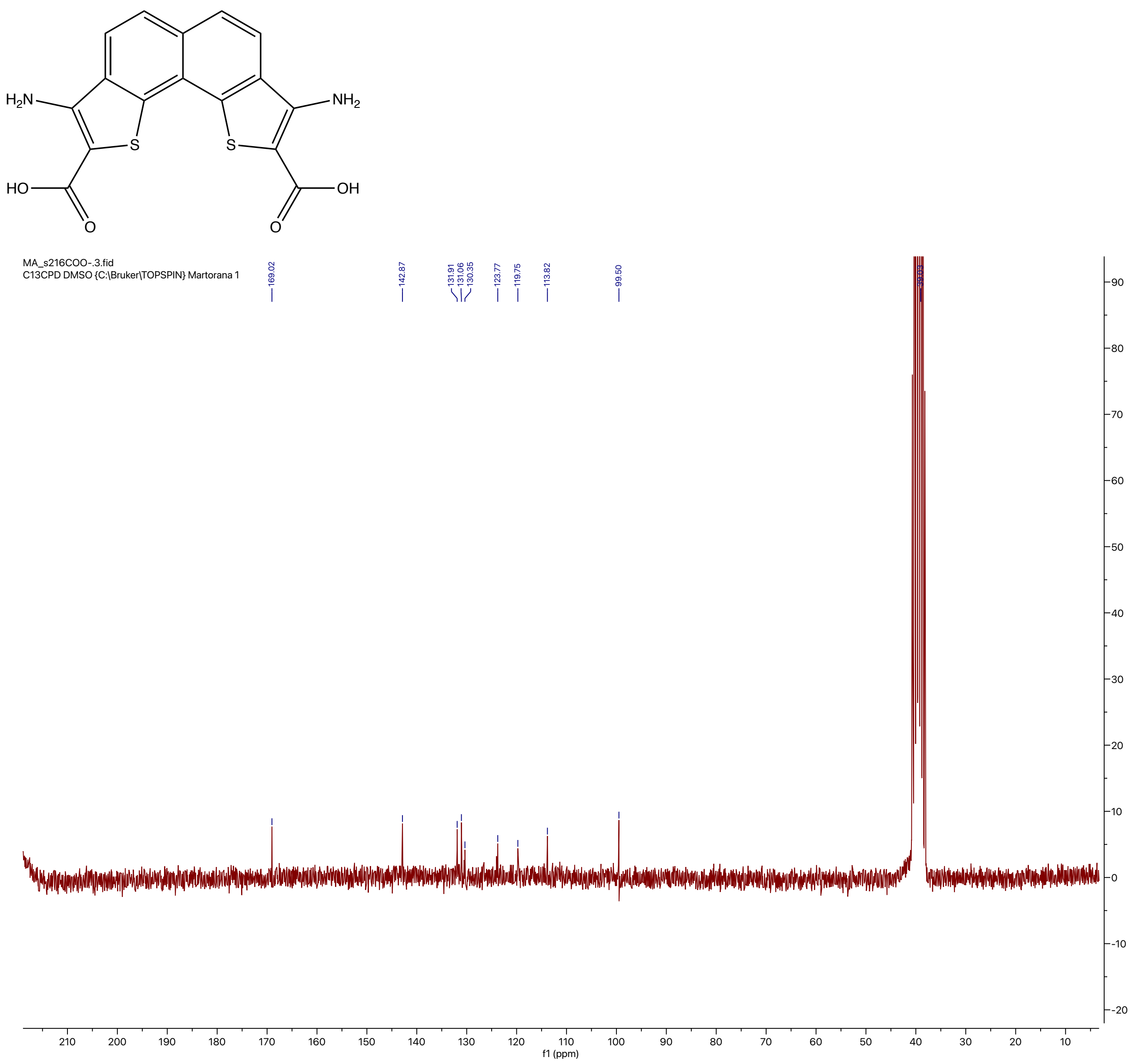

S5: ^{13}C NMR spectrum of 3,8-diamino-naphtho[1,2-b:8,7-b′]dithiophene-2,9-dicarboxylic acid **3**.

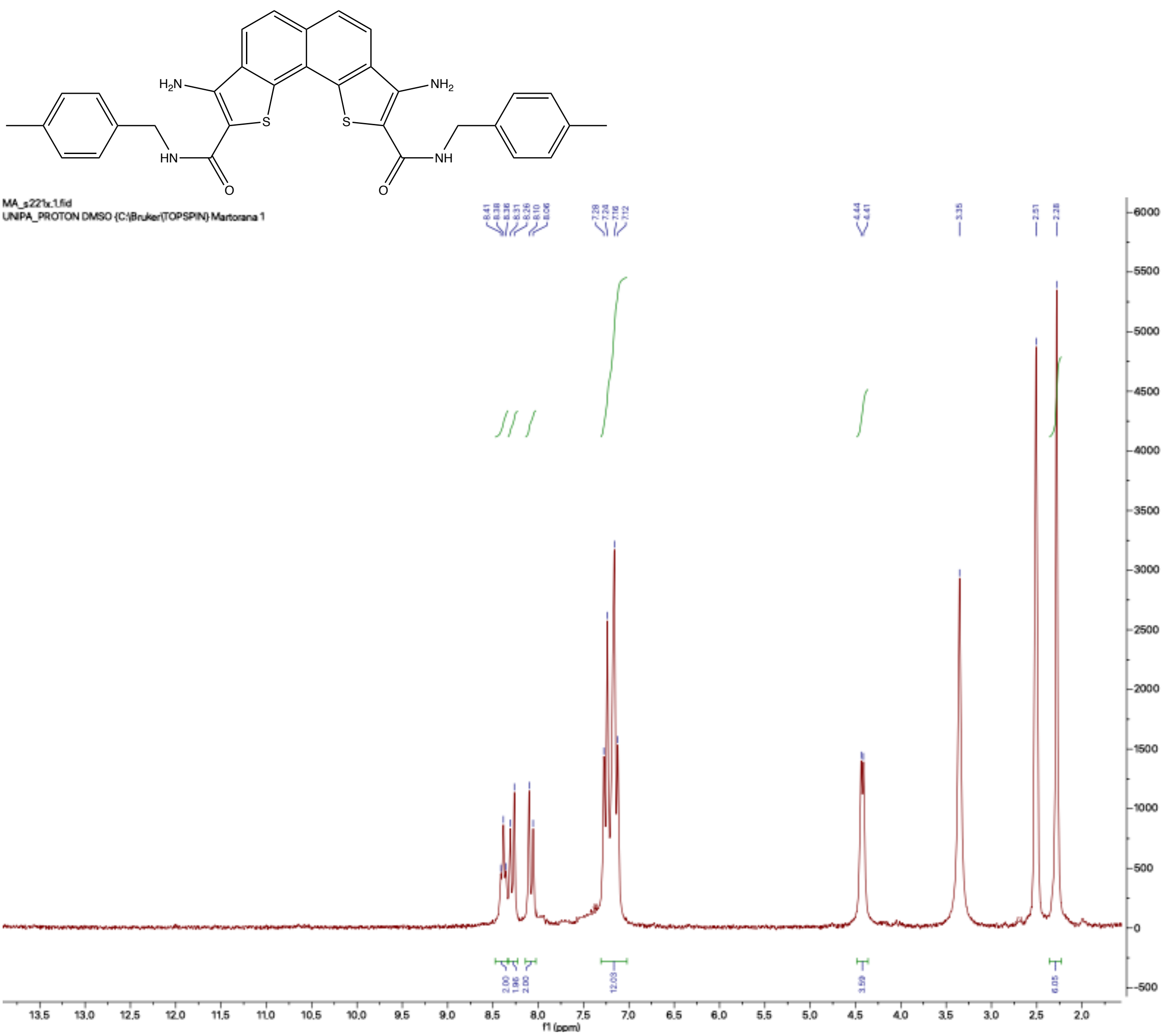

S6: ^{1}H NMR spectrum of 3,8-diamino-*N*[2],*N*[9]-bis(4-methylbenzyl)naphtho[1,2-*b*:8,7-*b*′]dithiophene-2,9-dicarboxamide **4a**.

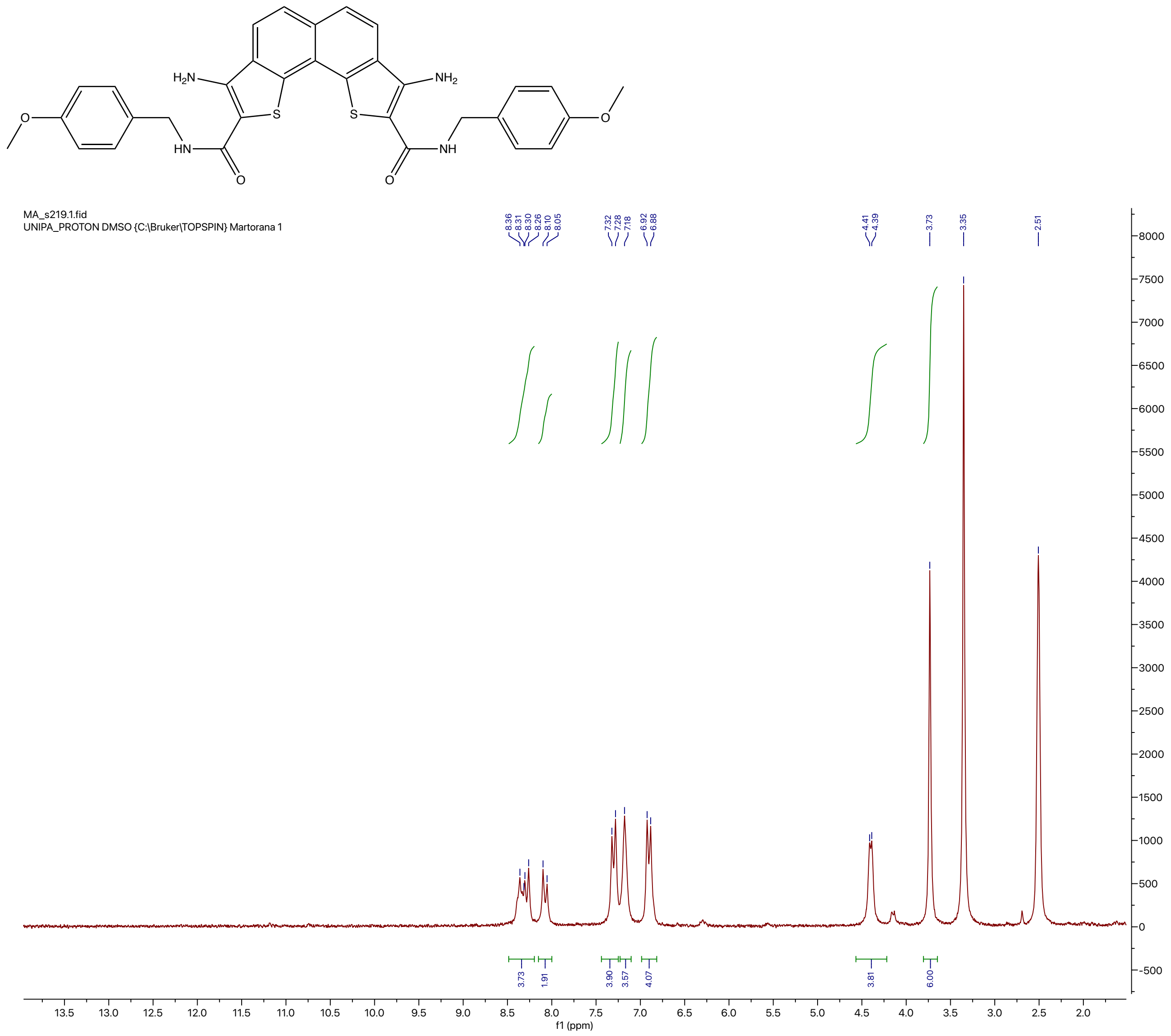

S7: ^{1}H NMR spectrum of 3,8-diamino-*N*[2],*N*[9]-bis(4-methoxybenzyl)naphtho[1,2-*b*:8,7-*b*′]dithiophene-2,9-dicarboxamide **4b**.

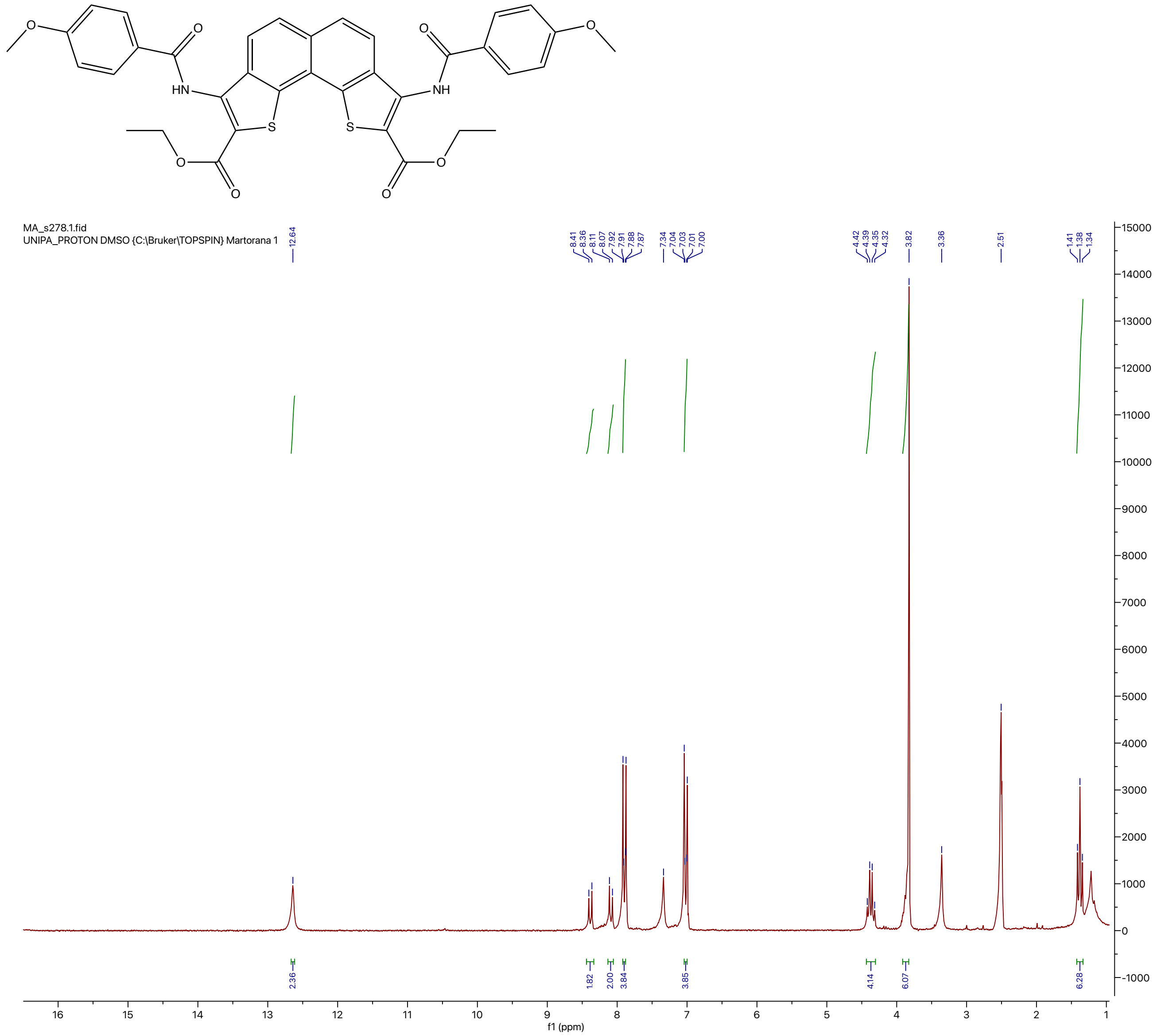

S8: ^{1}H NMR spectrum of diethyl 3,8-bis(4-methoxybenzamido)naphtho[1,2-*b*:8,7-*b′*]dithiophene-2,9-dicarboxylate **5b**.

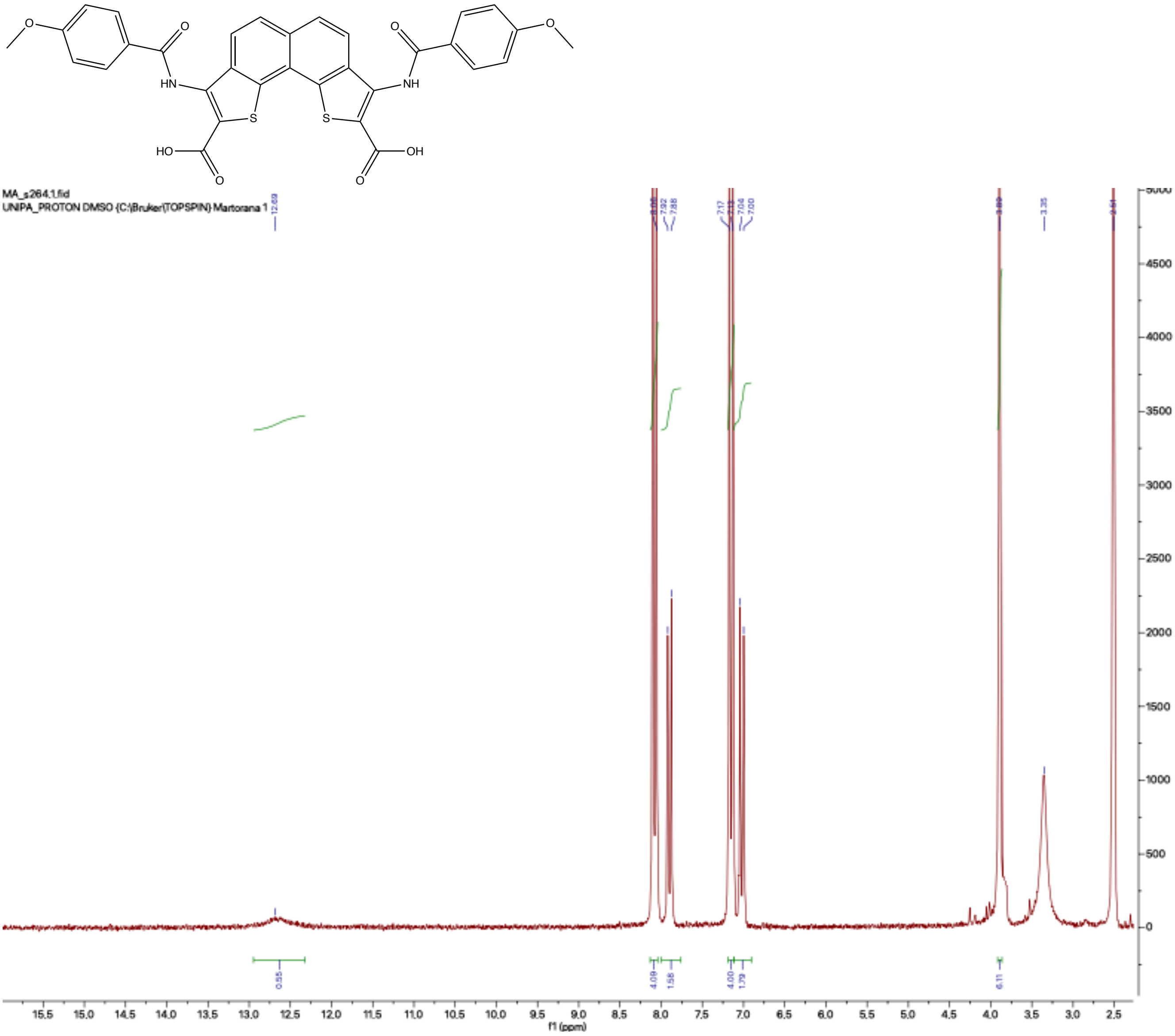

S9: ^{1}H NMR spectrum of 3,8-bis(4-methoxybenzamido)naphtho[1,2-*b*:8,7-*b′*]dithiophene-2,9-dicarboxylic acid**6b**.
